# Environment sustainability with smart grid sensor

**DOI:** 10.3389/frai.2024.1510410

**Published:** 2025-02-19

**Authors:** Sheetal Mahadik, Madhuri Gedam, Deven Shah

**Affiliations:** ^1^Department of Electronics and Telecommunication, Shree L.R. Tiwari College of Engineering, Thane, India; ^2^Department of Information Technology, Shree L.R. Tiwari College of Engineering, Thane, India

**Keywords:** smart grid, generation, distribution, consumption, communication, digital technology, renewable sources

## Abstract

Environmental sustainability is a pressing global concern, with energy conservation and efficient utilization playing a key role in its achievement. Smart grid technology has emerged as a promising solution, facilitating energy efficiency, promoting renewable energy integration, and fostering consumer engagement. But the addition of intelligent sensors to these grids has the potential to greatly increase the level of sustainability initiatives. *This paper highlights the role of smart grid sensors in addressing challenges like energy losses, demand-response limitations, and renewable energy integration. It explains how these sensors enable real-time monitoring, fault detection, and optimal load management to improve grid performance and reduce environmental impact.* This also study looks at how AI with smart grid sensor can perform real-time data monitoring, optimal energy distribution, and proactive decision support from smart grid sensors might improve environmental sustainability. *Furthermore, it examines advancements in sensor technologies in India, including pilot projects like the BESCOM initiative in Bangalore and Tata Power-DDL’s renewable energy trading in Delhi, to showcase their practical applications and outcomes.* Smart sensors provide accurate tracking of energy usage trends, enhance load distribution, and advance the sensible application of renewable energy resources. These sensors aid in cutting down on energy waste and carbon emissions by interacting with customers and enabling demand-response systems. This study addresses the critical role of smart sensors in overcoming the shortcomings of conventional grids and guaranteeing a more resilient, efficient, and sustainable energy future through an extensive analysis of the literature. Grid-enabled systems, such as electric water heaters with sensor, can achieve energy savings of up to 29%. The integration of renewable energy sources through sensors enhances system efficiency, reduces reliance on fossil fuels, and optimizes supply and demand. Utilizing Internet of Things (IoT) technology enables precise monitoring of air quality, water consumption, and resource management, significantly improving environmental oversight. This integration can lead to a reduction in greenhouse gas emissions by up to 20% and water usage by 30%. *Lastly, the paper discusses how integrating artificial intelligence with smart grid sensors can enhance predictive maintenance, energy management, and cybersecurity, further strengthening the case for their deployment.*

## 1 Introduction

The necessity to address climate change and the growing worldwide demand for energy have made environmental sustainability a top priority for all sectors of the economy. Energy systems play a key role in this shift, and smart grid technology is becoming increasingly important. a way to promote sustainable consumption habits, integrate renewable energy sources, and increase energy suppliers and consumers, and address these concerns (Caputo et al., 2018). Grid-enabled systems—such as electric water heaters—have shown energy savings of up to 29% (Booysen and Cloete, 2016). Additionally, sensors are essential to the integration of renewable energy sources since they control the erratic character of resources like solar and wind power, increasing system efficiency and lowering dependency on fossil fuels (Gantenbein et al., 2012; Keyhani and Marwali, 2012) and supply and demand while conserving energy (Arif, 2013). By providing accurate data gathering on air quality, water consumption, and resource management, the integration of Internet of Things (IoT) technology significantly improves environmental monitoring (Santosh et al., 2024). For instance, through enhanced energy use and resource management, smart grids can reduce greenhouse gas emissions by up to 20% and water usage by 30% (Nikolay et al., 2024).

Despite these promising benefits, the implementation of smart grid sensors is not without challenges. The effective implementation of these systems is hampered by problems including cybersecurity threats, the requirement for a strong infrastructure, and unpredictable environmental conditions (Belyakov et al., 2019). Furthermore, the increasing amount of data gathered from defect detection and real-time monitoring necessitates complex data processing skills, which could put a strain on current infrastructures (Slootweg et al., 2011). In order to fully utilize smart grid sensors for improving environmental sustainability, several issues must be resolved.

This paper aims to provide a comprehensive review of the literature on smart grid sensors’ role in promoting environmental sustainability. It explores key technological approaches such as real-time monitoring, optimized control models, and fault detection systems while analyzing the critical barriers to their successful implementation.

## 2 Smart grid sensor technology and sustainability

Smart grid sensors play a pivotal role in advancing environmental sustainability by driving energy efficiency, renewable energy integration, demand-side management, and consumer empowerment. These sensors significantly improve energy efficiency by enabling real-time monitoring and optimization of energy flows, which helps reduce transmission losses and minimizes energy waste, thereby lowering the carbon footprint (Chen et al., 2021). Additionally, they facilitate the seamless integration of renewable energy sources such as solar and wind into the grid. By dynamically adjusting energy distribution to accommodate the variability of these sources, smart grids ensure a stable and reliable energy supply while reducing reliance on non-renewable resources, contributing to a more sustainable energy system (Jang et al., 2021).

Moreover, smart grid sensors enhance demand response and load management capabilities, allowing the grid to adjust energy supply in real time based on consumption patterns. This not only reduces overproduction and minimizes energy wastage but also ensures a more sustainable and efficient use of resources (Nguyen and Nguyen, 2023). Furthermore, these technologies empower consumers by providing them with real-time insights into their energy usage, encouraging informed decision-making and fostering sustainable consumption practices (Khan et al., 2023).

## 3 Smart grid sensors

In order to improve power systems’ resilience, efficiency, and dependability, smart grid sensors are necessary. They support a number of technologies intended for real-time electrical grid management, optimization, and monitoring. The main categories of smart grid sensors that have been found in recent studies are described in the section that follows, along with their developments, especially in relation to India. Advancements in smart grids, including the integration of components such as AI and IoT for fault detection and energy optimization (Sandhya et al., 2024), smart metering for consumer engagement and energy conservation (Sanjay and Gupta., 2022), and the incorporation of renewable energy sources to enhance sustainability (Mythreyee and Nalini, 2024), offer transformative potential. The utilization of sensors and real-time data further optimizes energy distribution and minimizes outages (Sanjay and Gupta., 2022). However, while these developments highlight significant progress, recent innovations such as edge computing sensors, advanced fault detection mechanisms, and low-power wide-area network (LPWAN)-enabled devices remain underexplored.

Types of smart grid sensors:

1. Device and asset sensors: These sensors monitor critical components of the grid, including transformers, solar panels, and batteries. They provide essential data related to electrical, mechanical, and chemical properties, which helps in efficient asset management and system optimization (Mohsenian-Rad, 2022).
2. Building sensors: In smart buildings, occupancy, temperature, and illuminance sensors are deployed to manage and optimize energy consumption. These sensors contribute to more energy-efficient building designs by monitoring environmental conditions and adjusting energy use accordingly (Mohsenian-Rad, 2022).
3. Textile sensors: A novel advancement in smart grid technology includes textile-based sensors that can self-generate power. These sensors are capable of measuring both dynamic and static loads, enabling efficient energy use in various grid applications.

## 4 Smart grid sensor technologies in India

Smart grid technology plays a pivotal role in promoting environmental sustainability by addressing critical challenges in India’s power sector, such as high distribution losses, electricity theft, and environmental degradation. Key sensor technologies that have been created or integrated into India’s smart grid infrastructure include the following:

1. Wireless sensor networks (WSNs): These networks provide real-time monitoring and optimization of power transmission through the use of smart wireless transformer sensor nodes and consumer sensor nodes. WSNs help lower transmission losses and increase energy efficiency by providing continuous data gathering and communication (Devidas and Ramesh, 2010).
2. Advanced protection sensors: Protection sensors that can instantly modify their settings in response to the state of the electrical grid are among the latest developments in smart grid technology. Compared to conventional systems, these sensors provide up to an 80% boost in efficiency for defect detection (Alonso et al., 2020).

Additionally, case studies from Indian smart grid pilot projects, such as those implemented in Puducherry and Karnataka, could provide valuable insights into practical applications. Addressing these gaps is essential for leveraging smart grid technology to achieve long-term environmental sustainability in India. The National Smart Grid Mission (NSGM) drives smart grid deployment across India to enhance power grid efficiency and reliability. Pilot projects in Bangalore and Delhi showcase innovative applications. In Bangalore, BESCOM’s smart grid project with IIT and Mindteck focused on advanced metering and demand response to improve energy efficiency. In Delhi, Tata Power-DDL implemented a peer-to-peer solar energy trading pilot and a Battery Energy Storage System (BESS) project with the Asian Development Bank, demonstrating renewable energy integration and grid resilience. These initiatives highlight the potential of smart grid technologies for India’s energy future.

## 5 Challenges faced in smart grid sensor implementation

Although energy efficiency and grid reliability have significantly improved in India as a result of the development and implementation of smart grid sensors, a number of issues still need to be resolved. To the fullest extent possible, smart grid innovations cannot be realized without addressing these obstacles, which cut across the technological, economic, and regulatory sectors. In order to develop India’s energy infrastructure and promote a more sustainable and effective power system, these obstacles must be overcome.

The *conventional power grid* faces several challenges that hinder its efficiency and reliability. One of the major issues is the *inefficiency of energy transmission*, where significant energy losses occur over long distances due to outdated infrastructure (Zhao et al., 2021). Moreover, traditional grids struggle with *fault detection and recovery*, often relying on manual inspections to identify and fix problems, leading to longer downtimes (Khan et al., 2022). *Integrating renewable energy* sources, such as solar and wind, is also problematic due to their intermittent nature, making it difficult for conventional grids to maintain stability (Wang and Li, 2020). Furthermore, traditional grids lack the ability to dynamically manage *demand response*, resulting in grid congestion and inefficient resource use during peak demand periods (Wang and Li, 2020). The *aging infrastructure* of conventional grids adds to the problem, with many systems requiring expensive and time-consuming upgrades (Tan et al., 2021). Additionally, conventional grids are vulnerable to both *cyberattacks and physical damage*, primarily because of their centralized design and lack of advanced security measures (Li et al., 2022).

### 5.1 Technical challenges

1. Integration of renewable energy: The variability of renewable energy sources, such wind and solar power, makes energy system stability and management more difficult. Because these resources are unpredictable, sophisticated algorithms and real-time control systems are required to guarantee the steady and dependable integration of renewable energy into the grid (Saha et al., 2023).
2. Cybersecurity risks: Smart grids are more susceptible to cyberattacks since they primarily rely on digital technologies for data interchange, real-time monitoring, and control. Maintaining grid stability and safeguarding sensitive data requires securing the grid from illegal access and data breaches (Alhariry et al., 2021; Archana and Singh, 2022).

### 5.2 Economic challenges

1. High initial costs: Smart grid infrastructure implementation necessitates large upfront costs for sensors and smart meters. There is pressure to keep energy prices reasonable, but these high costs present a financial problem, especially in areas with low support (Archana and Singh, 2022). An ongoing economic problem is striking a balance between the need for affordable energy and investments in advanced technologies.
2. Lack of government policies and incentives: Smart grid implementation is slowed by the lack of comprehensive regulatory frameworks and government incentives. Consumer engagement is hampered and private sector investment is discouraged by insufficient policies promoting the integration of renewable energy sources and the development of smart grids (Dasari and Kaluri, 2024). Encouraging public-private collaborations and promoting the wider deployment of smart grid solutions require effective legislation.

### 5.3 Social and regulatory challenges

Consumer engagement and awareness: Active customer participation is essential to the success of smart grids, especially in demand-response schemes. Nevertheless, consumer participation is restricted by a widespread lack of knowledge and comprehension of the advantages of smart grids, which lowers the technology’s overall efficacy (Dasari and Kaluri, 2024). It is essential to address these technological, financial, and legal obstacles if smart grid sensors are to be successfully of smart grids and secure a future in energy that is more robust, efficient, and sustainable by removing these obstacles.

*Smart grid sensors* address these challenges by enabling a more efficient, reliable, and secure grid system. Smart grids use *real-time monitoring* to gather data on grid conditions, which facilitates immediate detection of faults and enhances the overall grid management (Garg et al., 2022). This *improved fault detection and automation* reduces downtime by isolating faults and rerouting power to unaffected areas, thereby speeding up recovery (Hassan et al., 2023). Additionally, *smart sensors enable better integration of renewable energy*, as they help to balance the supply and demand of energy through advanced algorithms, ensuring a steady flow of power even when renewable sources fluctuate (Jang et al., 2021). With smart grid sensors, *demand response and load management* become more efficient, as utilities can adjust energy distribution based on real-time usage data, minimizing congestion and optimizing resource distribution (Nguyen and Nguyen, 2023). The *enhanced energy efficiency* provided by smart sensors helps to identify and eliminate energy losses during transmission, which improves the overall performance of the grid (Chen et al., 2021). Moreover, smart grids offer *greater security* due to their distributed nature, allowing for quicker identification and mitigation of potential cyber threats or physical disruptions (Li et al., 2023). Finally, *consumer empowerment* is a notable benefit, as smart grids provide consumers with detailed usage data, enabling them to make informed decisions about energy consumption, resulting in cost savings and environmental benefits (Khan et al., 2023).

## 6 Integration of smart grid sensors through AI

Artificial intelligence (AI) with Smart Grid sensor integration offers revolutionary possibilities for improving the sustainability, dependability, and efficiency of contemporary energy systems. Smart grids can use AI technology to manage distributed energy resources (DERs) more effectively and optimize real-time operations, which will ultimately increase the stability and resilience of the grid as a whole. Smart sensors in smart grid enhance environmental monitoring by tracking parameters such as temperature, humidity, and air quality, enabling proactive responses to environmental changes (Caputo et al., 2018). Safety applications, such as detecting partial discharges in power devices, further underscore their utility in preventing energy losses and enhancing grid reliability (Booysen and Cloete, 2016). Additionally, intelligent resource management using technologies like fuzzy inference systems highlights their ability to optimize energy consumption and user comfort (Gantenbein et al., 2012). Integrating AI with smart sensor systems can amplify these benefits by enabling predictive analytics, automated fault detection, and secure communication, addressing critical challenges like data security and system integration (Keyhani and Marwali, 2012).

AI-driven smart sensors represent a transformative integration of artificial intelligence (AI) with sensing technologies, enhancing their accuracy, sensitivity, and adaptability across diverse fields, including industrial automation, agriculture, and biomedical engineering. These advancements leverage AI algorithms to improve sensor design, calibration, and object recognition, significantly boosting performance in applications such as imaging and environmental monitoring. The development of flexible sensors powered by AI has enabled efficient data acquisition and analysis, fostering innovations in robotics and human activity monitoring. In industrial contexts, virtual sensors, which serve as digital representations of physical devices, facilitate scalable and robust data generation, essential for predictive maintenance and anomaly detection in Industry 4.0 applications (“Virtual Sensors for Smart Data Generation and Processing in AI-Driven Industrial Applications,” 2023). Additionally, the advent of self-powered systems, such as those utilizing triboelectric nanogenerators, ensures sustainability while providing real-time monitoring and decision-making capabilities in smart industries. In agriculture, the integration of AI with bio-sensors optimizes crop management by analyzing soil and environmental parameters, addressing labor shortages, and improving food security.

1. Enhanced monitoring and control
  - Machine learning for load balancing: Smart grids can analyze real-time fluctuations in supply and demand to dynamically regulate energy distribution through sophisticated machine learning algorithms. Because less waste is generated during peak and off-peak hours, effective load balancing not only increases grid stability but also improves energy efficiency (Devidas and Ramesh, 2010).
2. Improved energy management
  - Renewable energy integration: Artificial Intelligence is a vital component in helping to integrate renewable energy sources. Artificial intelligence (AI) maximizes the use of resources by offering precise forecasting of energy generation from sporadic sources like solar and wind, enhancing overall system resilience. This lessens reliance on fossil fuels and helps create a more stable and sustainable energy landscape (Alonso et al., 2020).

## 7 Real time data

Practical implementation strategies and real-world case studies from existing literature demonstrate how AI-driven smart grids have been implemented effectively and offer insights into overcoming challenges. Here are some examples:

1. Real-time load balancing and demand response programs.
  Case study: The Pacific Northwest National Laboratory (PNNL) in the United States deployed an AI-based demand response system in partnership with regional utilities to manage peak load. Through machine learning algorithms, the system optimized energy distribution by adjusting appliance use during peak hours without compromising consumer comfort. This approach reduced energy costs and contributed to grid stability, showcasing how AI can enable responsive, data-driven load management.
  Strategy: Implementing AI-based load balancing requires predictive models that can anticipate demand and adjust supply accordingly. Utilities need to gather large datasets on consumption patterns, ideally from smart meters, to train algorithms and continuously refine accuracy through machine learning.
2. Predictive maintenance and fault detection.
  Case study: In Spain, Red Eléctrica, the country’s electricity grid operator, uses AI-based sensors and IoT devices for predictive maintenance. These technologies detect and predict potential faults within the grid, minimizing outages and extending the lifespan of equipment. Red Eléctrica has reported a significant decrease in operational costs due to this predictive maintenance approach, and the early fault detection has improved service reliability.
  Strategy: Implementing predictive maintenance requires high-quality sensor data from various grid components. Advanced AI algorithms analyze this data to detect anomalies, helping utilities address issues before they become critical. Building a dedicated data processing infrastructure is essential to manage and analyze large volumes of data.
3. Renewable energy integration with AI forecasting.
  Case study: In Germany, Tennet TSO, an energy company, deployed AI-based forecasting to better manage the integration of wind and solar energy into the grid. Their collaboration with IBM’s Watson allowed Tennet to predict renewable energy generation and adjust grid operations in real-time, stabilizing the grid and maximizing the use of clean energy sources.
  Strategy: Effective AI integration for renewable energy forecasting involves data collection on weather, demand patterns, and energy production from renewable sources. Algorithms use this data to predict output, enabling utilities to plan for fluctuations and reduce reliance on backup fossil fuel plants.
4. Cybersecurity measures in AI-powered grids.
  Case study: In Singapore, SP Group, the country’s electricity and gas distributor, implemented an AI-driven cybersecurity framework to secure its smart grid from cyber threats. By integrating machine learning algorithms to detect unusual patterns, SP Group mitigated potential cyber risks, ensuring secure, continuous operation of the smart grid.
  Strategy: Cybersecurity for AI-powered smart grids requires robust encryption, anomaly detection, and real-time monitoring systems. Utilities must also invest in training and regulatory compliance to ensure personnel can respond to detected threats swiftly.

These cases highlight the importance of targeted implementation strategies, robust data management, continuous model refinement, and proactive cybersecurity for AI-driven smart grids. By learning from these real-world applications, stakeholders can effectively address technological, economic, and regulatory challenges to leverage AI for sustainable and resilient energy systems.

## 8 Future direction

The future of AI-integrated smart grid sensors lies in advancing energy efficiency, reliability, and environmental sustainability by leveraging cutting-edge technologies. The integration of AI with smart grids enables real-time data analysis, predictive maintenance, and optimized energy management, which are essential for addressing dynamic energy demands and promoting the efficient distribution of resources. AI-driven algorithms facilitate enhanced energy management through sophisticated demand-response strategies, enabling grids to adapt effectively to consumption fluctuations. Techniques such as Non-Intrusive Load Monitoring (NILM), powered by AI, further improve energy optimization by analyzing usage patterns without requiring intrusive sensors.

Additionally, AI plays a pivotal role in integrating renewable energy into the grid by improving forecasting accuracy and optimizing generation processes, ensuring a seamless and sustainable energy mix. The synergistic application of AI and hydrogen energy systems is expected to transform energy distribution and utilization, supporting eco-friendly practices. However, challenges such as data privacy and security necessitate the development of robust protocols and standards for secure AI applications in smart grids.

To overcome current limitations, future research should focus on decentralized control mechanisms and enhancing interoperability between various technologies. Addressing these challenges, alongside regulatory barriers and technological constraints, is critical to fully unlocking the potential of AI-integrated smart grids in achieving environmental sustainability.

## 9 Conclusion

When paired with AI technology, smart grid sensors have the ability to completely transform energy management by building a power infrastructure that is dependable, efficient, and focused on the needs of its customers. By enhancing the integration of renewable energy, lowering transmission losses, and encouraging more active consumer participation in energy-saving initiatives, these advances have the potential to dramatically enhance environmental sustainability. *The paper highlights how smart grid sensors, such as current and asset sensors, contribute to real-time fault detection, load optimization, and improved energy efficiency. These advancements reduce dependency on fossil fuels while enabling better demand-response mechanisms to stabilize energy supply and consumption.* But in order to fully profit from smart grids, a number of technological, financial, and legal obstacles must be overcome. *One critical challenge is the high initial cost of implementing sensor infrastructure, which requires government support and incentives to encourage adoption. Furthermore, addressing cybersecurity threats is essential to safeguard sensitive energy data and ensure grid stability.* To speed up the construction of infrastructure, governments must enact laws that encourage public-private partnerships and investment in smart grid technologies. *The successful integration of AI-driven technologies, such as predictive maintenance systems and renewable energy forecasting tools, has been demonstrated through case studies from India and abroad. These examples underscore the importance of leveraging AI for real-time data analysis, enabling utilities to address operational inefficiencies proactively.* Furthermore, overcoming technological obstacles like cybersecurity issues and the integration of renewable energy sources will require constant research and innovation. In the end, smart grid sensors are an important technological enabler for a future with more sustainable energy sources. The strategic deployment of smart grid sensors combined with AI integration can assist meet India’s energy needs while encouraging long-term environmental and economic sustainability as the nation continues to update its energy infrastructure and shift to greener power sources. *As highlighted in the paper, initiatives like the National Smart Grid Mission (NSGM) and pilot projects in Bangalore and Delhi demonstrate the feasibility and benefits of advanced smart grid technologies. These efforts should be expanded and replicated to create a resilient energy network that meets both urban and rural demands.* Unlocking the full potential of these technologies and assuring a more robust, efficient, and sustainable energy system for future generations will require addressing the implementation-related problems.
